# Antibody Complementarity-Determining Regions (CDRs) Can Display Differential Antimicrobial, Antiviral and Antitumor Activities

**DOI:** 10.1371/journal.pone.0002371

**Published:** 2008-06-11

**Authors:** Luciano Polonelli, José Pontón, Natalia Elguezabal, María Dolores Moragues, Claudio Casoli, Elisabetta Pilotti, Paola Ronzi, Andrey S. Dobroff, Elaine G. Rodrigues, Maria A. Juliano, Domenico Leonardo Maffei, Walter Magliani, Stefania Conti, Luiz R. Travassos

**Affiliations:** 1 Dipartimento di Patologia e Medicina di Laboratorio, Sezione di Microbiologia, Università degli Studi di Parma, Parma, Italy; 2 Departamento de Inmunología, Microbiología y Parasitología, Facultad de Medicina y Odontología, Universidad del País Vasco, Bilbao, Vizcaya, Spain; 3 Departamento de Enfermería I, Universidad del País Vasco, Bilbao, Vizcaya, Spain; 4 Dipartimento di Scienze Cliniche L. Sacco, Sezione di Malattie Infettive e di Immunopatologia, Università di Milano, Milano, Italy; 5 Dipartimento di Clinica Medica, Nefrologia e Scienze della Prevenzione, Università degli Studi di Parma, Parma, Italy; 6 Unidade de Oncologia Experimental, Departamento de Microbiologia, Imunologia e Parasitologia, Universidade Federal de São Paulo, São Paulo, Brazil; 7 Departamento de Biofisica, Universidade Federal de São Paulo, São Paulo, Brazil; Cairo University, Egypt

## Abstract

**Background:**

Complementarity-determining regions (CDRs) are immunoglobulin (Ig) hypervariable domains that determine specific antibody (Ab) binding. We have shown that synthetic CDR-related peptides and many decapeptides spanning the variable region of a recombinant yeast killer toxin-like antiidiotypic Ab are candidacidal *in vitro*. An alanine-substituted decapeptide from the variable region of this Ab displayed increased cytotoxicity *in vitro* and/or therapeutic effects *in vivo* against various bacteria, fungi, protozoa and viruses. The possibility that isolated CDRs, represented by short synthetic peptides, may display antimicrobial, antiviral and antitumor activities irrespective of Ab specificity for a given antigen is addressed here.

**Methodology/Principal Findings:**

CDR-based synthetic peptides of murine and human monoclonal Abs directed to: a) a protein epitope of *Candida albicans* cell wall stress mannoprotein; b) a synthetic peptide containing well-characterized B-cell and T-cell epitopes; c) a carbohydrate blood group A substance, showed differential inhibitory activities *in vitro, ex vivo* and/or *in vivo* against *C. albicans*, HIV-1 and B16F10-Nex2 melanoma cells, conceivably involving different mechanisms of action. Antitumor activities involved peptide-induced caspase-dependent apoptosis. Engineered peptides, obtained by alanine substitution of Ig CDR sequences, and used as surrogates of natural point mutations, showed further differential increased/unaltered/decreased antimicrobial, antiviral and/or antitumor activities. The inhibitory effects observed were largely independent of the specificity of the native Ab and involved chiefly germline encoded CDR1 and CDR2 of light and heavy chains.

**Conclusions/Significance:**

The high frequency of bioactive peptides based on CDRs suggests that Ig molecules are sources of an unlimited number of sequences potentially active against infectious agents and tumor cells. The easy production and low cost of small sized synthetic peptides representing Ig CDRs and the possibility of peptide engineering and chemical optimization associated to new delivery mechanisms are expected to give rise to a new generation of therapeutic agents.

## Introduction

Immunoglobulins are composed of polymorphic heavy and light chains. The idiotypic variability is related to the diversity of the antigen binding site and in particular to the hypervariable domains called complementarity-determining regions (CDRs). There are 6 CDRs in both variable regions of light (V_L_) and heavy chains (V_H_) with background variability on each side of the CDRs. Antibodies (Abs) of different specificities can assemble identical V_L_ domains with different V_H_ domains. The framework sequences between CDRs can be similar or identical.

Idiotypic vaccination with a murine monoclonal Ab (mAb KT4), that neutralizes the wide-spectrum antimicrobial activity of a yeast killer toxin (KT) against eukaryotic and prokaryotic microorganisms presenting specific cell wall receptors (KTR), elicited the production of a special sub-set of antiidiotypic Abs (KT-antiId) characterized by *in vitro* microbicidal and *in vivo* therapeutic effects [1]. Abs functionally mimetizing KT were detected in the serum or secretions of animals and humans experimentally or naturally infected with KTR-bearing microorganisms (KT-Abs) and have been produced in the monoclonal (KT-mAb) and recombinant (KT-scFv) formats. KT-Abs, KT-mAb and KT-scFv conferred passive immunoprotection in experimental models of mucosal and systemic fungal infections [2]–[4].

The peptides corresponding to the CDRs of KT-scFv, as well as a series of two-residue displaced overlapping decapeptides spanning the variable region were synthesized. All the synthetic CDRs and most of the related decapeptides showed a fungicidal effect against *Candida albicans in vitro* [5]. The most *in vitro* and *in vivo* active fragment (P6), including seven amino acids of the framework region and the first three residues of the light chain CDR1 of KT-scFv, was well represented among the sequences of many unrelated Abs. A killer decapeptide (KP) generated by alanine substitution of the first aminoacid of P6 had increased candidacidal activity *in vitro*. Significantly, KP exerted therapeutic effect in well established murine models of vaginal and systemic candidiasis, cryptococcosis and paracoccidioidomycosis [5]–[7]. KP, KT-Abs, KT-mAb and KT-scFv exerted a broad microbicidal activity *in vitro* including pathogenic bacteria and protozoa [8], [9]. Surprisingly, based on the sequence homology of P6 with critical segments of the gp160 precursor, KP proved to inhibit the *in vitro* and *ex vivo* replication of HIV-1 [10].

A mAb (C7), raised against *C. albicans* cell wall stress mannoprotein, a major target of human secretory IgA in the course of oral and vaginal candidiasis, has been recently described [11]. As a polyreactive IgM, mAb C7 cross-reacted with cell wall proteins of *C. albicans* Als3 and enolase, as well as with the nuclear pore complex Nup88 [12], [13]. MAb C7 is the first Ab able to exert three different antifungal activities against *C. albicans*, such as inhibition of germination and adhesion and a direct fungicidal effect which extended to *C. lusitaniae*, *Cryptococcus neoformans*, *Aspergillus fumigatus*, and *Scedosporium prolificans*, suggesting a pleiotropic mode of action [11]. Recently, mAb C7 proved to be protective in a murine model of systemic candidiasis [14].

As a proof of concept of the extrinsic potential of Ab fragments, we now report on the differential antimicrobial, antiviral and antitumor activities of synthetic peptides with sequences identical to CDRs of the light chain (L1, L2 and L3) and heavy chain (H1, H2 and H3) of: a) mAb C7; b) mouse mAb pc42 (IgM), directed to a synthetic peptide containing the surface antigen of hepatitis B virus and the T-helper-cell epitope from the circumsporozoite protein of *Plasmodium falciparum*, sharing H1 and H2 with mAb C7 [15]; and c) a human IgM mAb (HuA) specific for difucosyl human blood group A substance sharing no CDR homology with either mAb C7 or mAb pc42 [16].

## Results

### Anticandidal activities of CDR-based synthetic peptides

**Figure 1:**
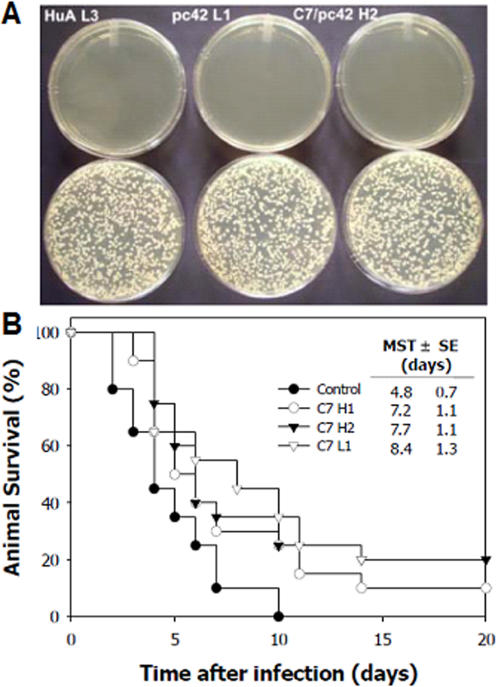
Anticandidal activities of mAb CDRs. A. *In vitro* candidacidal activity of three different CDR peptides (HuA L3, pc42 L1, C7/pc42 H2) against *Candida albicans* UP 10 cells as shown by CFU assay. *C. albicans* cells were treated with 100 µg/ml of each peptide in distilled water (*upper*) in comparison with distilled water only (*lower*). Each plate is representative of an assay carried out in triplicate; B. Effect of mAb C7/pc42 H1 and H2 and mAb C7 L1 CDRs on the survival curve of mice (11–12 animals/group) infected intravenously with 5×10^5^ yeast cells of *C. albicans*. The survival curve of mAb C7 L1-treated animals was significantly different (p  =  0.01) from that of control mice. MST±SE mean survival time±standard errors.

The *in vitro* microbicidal activity of mAb C7, mAb pc42 and mAb HuA CDR-based synthetic peptides at 100 µg/mL, and the EC_50_ of CDRs that exhibited a significant activity against *C. albicans* UP10 are shown in Table 1. The most active CDR peptides were mAb pc42 L1, mAb C7/pc42 H2 and HuA L3 (Fig. 1A). Similar results were obtained with *C. albicans* NCPF 3153 (data not shown). Most H2 alanine-substituted derivatives (*asd*) had activities similar to H2, but some showed modulating effects (Table 2). Substitution of the last residue in H2 (K16A) resulted in a fundamental loss of activity whereas substitution of the previous amino acid (F15A) gave rise to the highest candidacidal derivative.

**Table 1:** *In vitro* microbicidal activity of mAb C7, mAb pc42 and mAb HuA CDRs tested as synthetic peptides against *Candida albicans.*

mAb CDR	Anti-*Candida* activity	
	(%) at 100 µg/mL	EC_50_ (95% confidence intervals) [mol/L]
C7 L1 KSSQSLLNSGNQKNYLT	100	1.523 (1.123–2.066)×10^−5^
C7 L2 WASTRES	49.8	
C7 L3 NDYSYPRSR	99.3	2.019 (1.270–3.206)×10^−5^
C7/pc42 H1 GYYMH	69.2	
C7/pc42 H2 YISCYNGATSYNQKFK	100	4.193 (3.416–5.146)×10^−6^
C7 H3 ARQGVRGGAMD	55.5	
pc42 L1 YRASKSVSTSGYSYMH	100	7.801 (7.401–8.223)×10^−7^
pc42 L2 LVSNLES	100	1.196 (1.115–1.283)×10^−5^
pc42 L3 QHIRELTRSE	83.9	
pc42 H3 PNPLKAM	25.9	
HuA L1 RASQSVSSYLA	0	
HuA L2 DASNRAT	0	
HuA L3 QQRSNWPRS	100	5.109 (3.408–7.662)×10^−6^
HuA H1 SYTFH	12.9	
HuA H2 VLAYDGSYQHYADSVKG	0	
HuA H3 GQTTVTKIDEDY	19.2	

**Table 2:** *In vitro* microbicidal activity of mAb C7/pc42 H2 alanine-substituted derivatives against *Candida albicans.*

*asd* 1	Anti-*Candida* activity		EC_50_asd/EC_50_H2
	(%) at 100 µg/mL	EC_50_ (95% confidence intervals) [mol/L]	
Y1A	99.37	6.212 (3.668–10.507)×10^−6^	1.48
I2A	99.77	6.249 (0.416–93.929)×10^−6^	1.49
S3A	100.00	3.115 (3.114–3.115)×10^−6^	0.74
C4A	94.51	8.226 (6.911–9.790)×10^−6^	1.96
Y5A	98.10	2.853 (2.836–2.870)×10^−5^	6.81
N6A	100.00	1.542 (1.345–1.767)×10^−6^	0.37
G7A	100.00	7.312 (7.148–7.485)×10^−6^	1.74
T9A	100.00	3.798 (3.659–3.942)×10^−6^	0.91
S10A	100.00	1.324 (1.317–1.330)×10^−5^	3.16
Y11A	55.70	4.759 (3.798–5.961)×10^−5^	11.35
N12A	100.00	6.480 (5.231–8.031)×10^−6^	1.55
Q13A	100.00	3.912 (3.343–4.577)×10^−6^	0.93
K14A	91.72	1.850 (1.005–3.405)×10^−5^	4.41
F15A	100.00	9.039 (8.729–9.353)×10^−7^	0.22
K16A	0	1.608 (1.205–2.144)×10^−4^	38.35

1 *asd*: alanine-substituted derivatives.

MAb C7/pc42 H1 and H2, and mAb C7 L1 CDRs also conferred protection against invasive candidiasis. Treatment of infected animals with the peptides led to increased survival time with some fully protected animals at the end of the experiment (Fig. 1B). The survival curves correlated well with differences in fungal burden in kidney tissue, particularly in animals treated with mAb C7 L1 CDR. On day 5 post-infection, the CFU counts in kidney tissue were 160.3±46.6, 53.4±52.0, 119.6±35.5, and 133.4±43.5 for mice treated with saline, mAb C7 L1, mAb C7/pc42 H1 and H2 CDRs, respectively. When compared with the results obtained in saline treated animals, only the results from mice treated with mAb C7 L1 were statistically significant (p = 0.02).

### Anti HIV-1 activity of CDR-based synthetic peptides

The *ex vivo* and *in vitro* activities of synthetic peptides corresponding to mAb C7, mAb pc42, mAb HuA CDRs and *asd* of mAb C7/pc42 H1 against HIV-1 are shown in Tables 3 and 4. The kinetics of viral Ag production in untreated cultures corresponded to 100% of viral production. Results are representative of 4 independent experiments performed for each assay condition. Percent values of HIV-1 inhibition express the mean of 3 determinations observed on day 10 of cultures. In *ex vivo* conditions, R5 HIV-1 replication in treated cultures was inhibited (>50%) by mAb C7/pc42 H1, and even more (about 90%) by mAb pc42 L1. In addition, a derivative of mAb C7/pc42 H1, H1 Y3A, had enhanced inhibitory effect on HIV-1, while G1A and H5A lost their activity, showing that substitution of one residue can influence the antiviral property. In experimental conditions allowing the infection of healthy PHA-activated PBMCs, the peptide-mediated effect was dependent on the HIV-1 phenotype. As shown in Table 3, by using the BaL strain (R5) results similar to the ones of the endogenous replication model were obtained. In contrast, except for mAb C7 H3, all peptides analyzed using IIIB strain (X4) were unable to block viral replication. These results provide evidence that CDR-based synthetic peptides may exert a potent control over R5 HIV-1 replication. They also point to a difference in the biologic properties of the peptides in relation to the HIV-1 viral strain used and probably also to the activation state of PBMCs.

**Table 3:** *Ex vivo* and *in vitro* inhibitory activity (%) of synthetic mAb C7, mAb pc42, and mAb HuA CDRs against HIV-1.

mAb CDR	% inhibitory activity (10 µg/ml)		
	endogenous HIV-1 replication	exogenous HIV-1 replication	
		BaL	IIIB
C7 L1	37	9	0
C7 L2	38	59	4
C7 L3	49	18	0
C7/pc42 H1	58	24	0
C7/pc42 H2	18	0	0
C7 H3	0	0	75
pc42 L1	91	41	0
pc42 L2	0	0	0
pc42 L3	0	0	0
pc42 H3	0	11	0
HuA L1	48	15	0
HuA L2	32	0	0
HuA L3	70	63	0
HuA H1	38	84	0
HuA H2	40	74	0
HuA H3	36	0	0

**Table 4:** *Ex vivo* inhibitory activity (%) of synthetic mAb C7 CDR H1 alanine-substituted derivatives against HIV-1.

mAb C7/pc42 H1 *asd* 1	% inhibitory activity (10 µg/ml)
G1A	0
Y2A	61
Y3A	90
M4A	41
H5A	0

1 *asd*: alanine-substituted derivatives.

### Antitumor activities of CDR-based synthetic peptides

**Figure 2:**
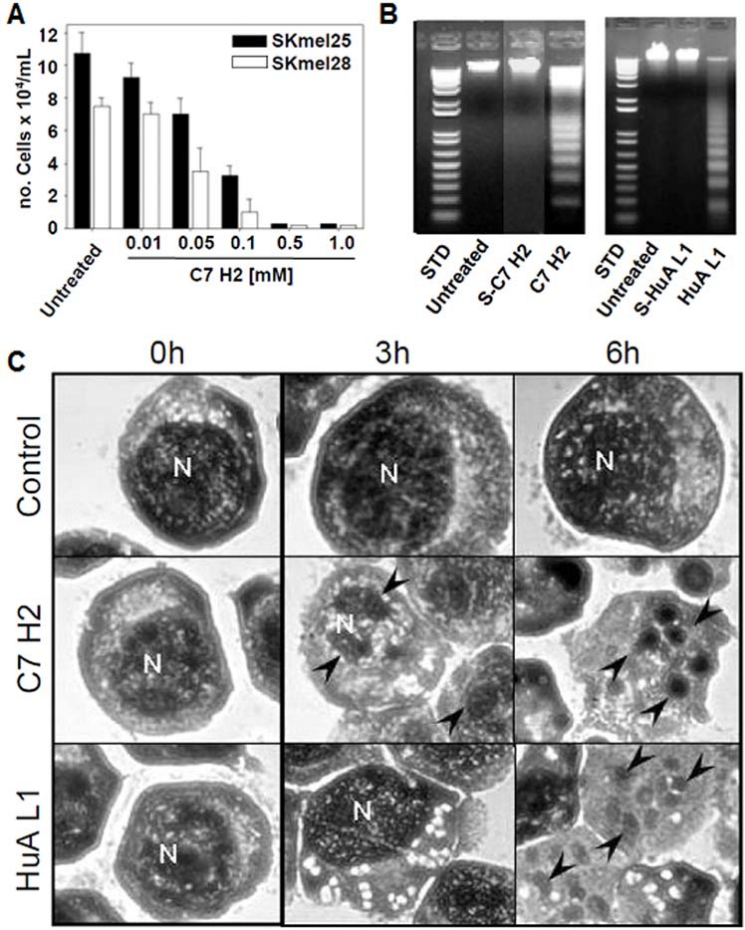
Antitumor effects of mAb CDRs. A. *In vitro* cytotoxic effects of C7/pc42 H2 in human melanoma cell lineages; B. DNA degradation in B16F10-Nex2 cells induced by CDRs C7/pc42 H2 and HuA L1; S-C7H2 and S-HuaL1, scrambled peptides; STD, 1 Kb Plus DNA Ladder, Invitrogen; C. Apoptotic response of HL-60 cells to peptides C7/pc42 H2 and HuA L1. HL-60 cells (5×10^5^) were treated with 0.5 mM of C7/pc42 H2 and HuA L1 peptides and incubated at 37°C. The material examined after 0, 3 and 6 h of incubation was collected by cytospin and stained (H&E). Peptide-treated cells are shown with altered morphology, surface blebs, and nuclear fragmentation (arrows). N =  cell nucleus; 100× magnification.

All CDR peptides from all three mAbs were assayed for cytotoxicity in B16F10-Nex2 murine melanoma cells. Although most of them were inactive, C7/pc42 H2 inhibited 50% of tumor cell growth at 0.05 mM; mAb HuA L1 was also effective but 5–10-fold less inhibitory than C7/pc42 H2 (Table 5). MAb C7/pc42 H2 was equally cytotoxic to human melanoma cell lines SKmel-25 and SKmel-28 (Fig. 2A). Both C7/pc42 H2 and HuA L1 caused DNA degradation in melanoma cells (Fig. 2B). The corresponding scrambled peptides were inactive. Additional data confirmed that both peptides were apoptotic not only in melanoma cells but also in HL-60 leukemia cells. Apoptosis was caspase-dependent and inhibitable by z-VAD, a pan-caspase inhibitor. Typical apoptotic alterations were seen in HL-60 cells with surface blebs and nuclear fragmentation (Fig. 2C).

**Table 5:** *In vitro* antitumor activity of selected synthetic mAb C7, mAb pc42, and mAb HuA CDRs against B16F10-Nex2 murine melanoma cells.

CDR	EC_50_ (95% confidence intervals) [mol/L]
mAb C7/pc42 H2	5.35 (2.220–8.480)×10^−5^
mAb HuA L1	7.20 (6.666–7.734)×10^−4^

C7/pc42 H2 peptide is predicted to contain a beta amphipathic (Eisenberg) region corresponding to SYNQKFK C-terminal sequence. Alanine substitutions in this region, rather than on the N-terminal (YISCYN) and turn region (GAT) reduced the cytotoxicity of H2 (Table 6).

**Table 6:** *In vitro* antitumor activity of synthetic mAb C7/pc 42 H2 alanine-substituted derivatives against B16F10-Nex2 murine melanoma cells.

mAb C7/pc42 *asd* 1	EC_50_ (95% confidence intervals) [mol/L]	EC_50_ *asd*/EC_50_H2
Y1A	7.14 (3.587–10.693)×10^−5^	1.33
I2A	7.35 (3.847–10.853)×10^−5^	1.37
S3A	6.49 (4.751–8.229)×10^−5^	1.21
C4A	6.75 (3.222–10.278)×10^−5^	1.26
Y5A	7.35 (3.773–10.927)×10^−5^	1.37
N6A	7.69 (5.951–9.429)×10^−5^	1.44
G7A	6.32 (2.817–9.823)×10^−5^	1.18
T9A	6.02 (2.517–9.523)×10^−5^	1.13
S10A	1.19 (1.016–1.364)×10^−4^	2.22
Y11A	1.13 (0.954–1.306)×10^−4^	2.11
N12A	1.25 (1.163–1.337)×10^−4^	2.34
Q13A	1.28 (0.922–1.638)×10^−4^	2.39
K14A	3.84 (3.654–4.026)×10^−4^	7.18
F15A	1.00 (0.982–1.018)×10^−3^	18.69
K16A	8.7 (5.073–12.327)×10^−5^	1.63

1 *asd*: alanine-substituted derivatives.

**Figure 3:**
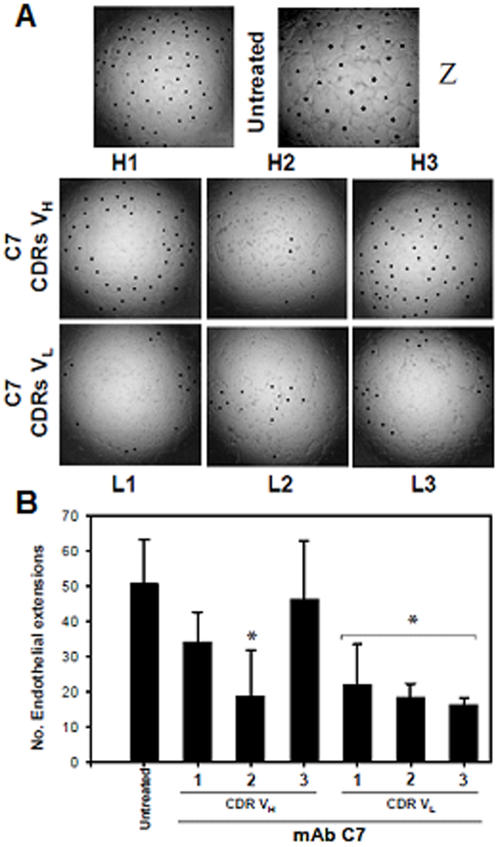
Angiogenesis inhibition by mAb CDRs. A. Inhibition of human endothelial cells (HUVEC) sprouting on Matrigel™ to form closed intercellular pro-angiogenic structures by mAb C7 CDRs; dots show individual rings. Untreated cell extensions are shown without and with zooming (Z); B. Counting of pro-angiogenic structures after 16 h incubation with peptides, in triplicate wells; *p< 0.01 relative to untreated control.

*In vivo* growth of tumor cells depends strictly on angiogenesis, therefore CDR peptides were also tested for endothelial cell cytotoxicity. The mAb C7 CDRs inhibited endothelial cell sprouting as tested *in vitro* with HUVEC cells grown on Matrigel™. CDRs H2, and also L1, L2 and L3 at higher concentrations, inhibited endothelial cell sprouting (Fig. 3A, B). These results show that CDRs from the same mAb, but mostly H2 have a capacity to inhibit both tumor cell growth and endothelial cell motility and tube formation.

**Figure 4:**
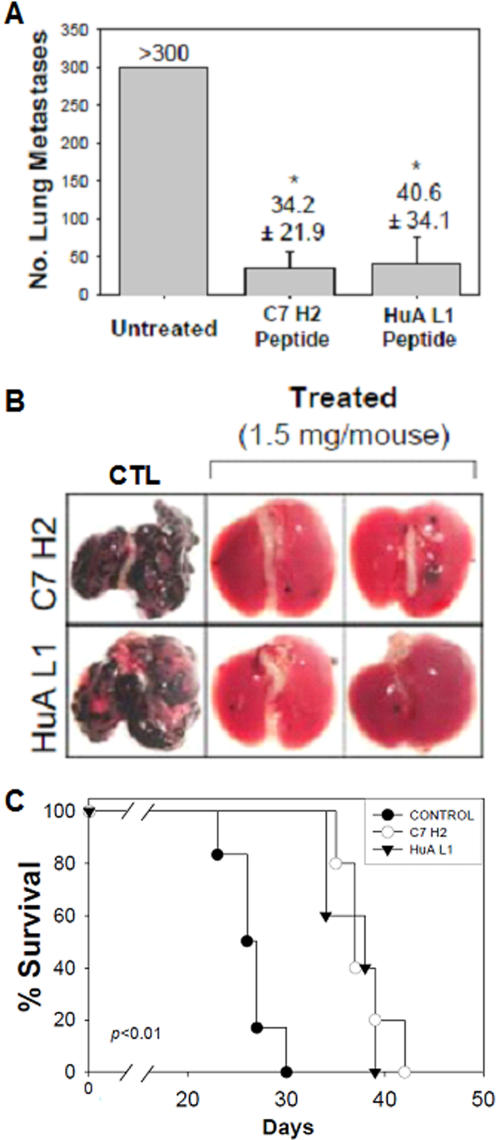
*In vivo* antitumor effects of two apoptotic mAb CDRs. A. *In vivo* protection (*p<0.001) by CDRs C7/pc42 H2 and HuA-L1 against lung colonization by B16F10-Nex2 melanoma cells (5×10^5^) injected i.v. in C57Bl/6 mice (10 animals/group). Peptides were administered (250 µg i.p.) on days 1, 3, 5, 7, 9, 11 after tumor cell challenge. B. Lungs of peptide-treated animals after 23 days of tumor challenge; CTL-control, untreated animals. C. Survival of peptide-treated mice and PBS controls with treatment being interrupted after day 11 of tumor cell i.v. challenge (p = 0.0014).

Administration of mAb C7/pc42 H2 and mAb HuA L1 intraperitoneally showed antitumor effects *in vivo* in a model of lung colonization by melanoma cells. While the untreated controls had >300 black nodules in the lungs as a result of intravenous injection of B16F10-Nex2 cells syngeneic to C57BL6 mice, the peptide-treated animals had lungs with 34–40 nodules. A significant increase in the survival time of peptide-treated animals was observed; mice died several days after peptide treatment was interrupted (Fig. 4A, B, C).

## Discussion

CDR sequences in the variable regions of immunoglobulins are thought to act cooperatively in the recognition of an antigen. Among them, CDR H3 is at the center of antigen recognition, but the other five CDRs are more or less involved for increased binding affinity to the antigen and some contact residues can even be located within framework regions [17]. The observation that Ab specificity is determined by a limited number of residues has allowed the synthesis of small peptides based on CDRs which retain binding properties and functions of the intact Ab [18], [19]. Diversity of CDR1 and CDR2 is encoded by the germline and further modified by somatic mutation; that of CDR L3 and CDR H3 arises somatically by rearrangement of the V segment with the JL or DH and JH segments, respectively.

Isolated CDR sequences, frequently CDR H3, showed the same specificity of the native Ab and were called micro(mini)antibodies [20], [21]. Diversity of CDR H3 is sufficient to allow otherwise identical IgM molecules to distinguish between a variety of haptens and protein antigens [22]. It has even been postulated that diverse CDR3 loops represent a highly antigen specific recognition core whereas other CDRs bind opportunistically [23]. Recently, a tyrosine-sulfated peptide derived from CDR H3 of an HIV-1-neutralizing Ab was shown to bind gp120 and inhibit HIV-1 infection [24]. Apart from the microantibodies that may display anti-viral [25] and antitumor [26] activities as does the native Ab, less is known about the biological activities of other CDR and framework sequences tested as isolated peptides. Some CDR and framework region-derived peptides, however, have been described as inhibitors of receptor-ligand interactions, cell adhesion and of microbial or viral infections [5], [27]. Several peptides including amino acids from the CDRs of anti-CD4 mAb ST40 and framework residues flanking the CDRs bound to soluble CD4 and displaced Ab binding [28]. Bioactive paratope-derived peptides of potential pharmacological interest were also deduced by hydropathic complementarity [29].

Here we show that, independent of the specificity of the native Ab, CDRs other than H3 may display, with high frequency, antimicrobial, antiviral and antitumor activities in a way reminiscent of molecules of early innate immunity [30]. Synthetic peptides representing the CDRs of a native Ab (mAb C7), raised against a *C. albicans* antigen, and also CDRs from mouse mAb pc42, sharing H1 and H2 with mAb C7, and human mAb HuA, sharing no CDR either with mAb C7 or mAb pc42, showed *in vitro*, *ex vivo* and/or *in vivo* differential antimicrobial, antiviral and/or antitumor activities.

The *in vivo* antitumor activity of mAb C7/pc42 H2 and mAb HuA L1, the protection conferred by mAb C7/pc42 H1, H2 and mAb C7 L1 against invasive candidiasis, as well as *ex vivo* inhibitory activity of mAb pc42 L1 against HIV-1 are examples of hypervariable Ab sequences with biological activity. Synthetic peptides representing Ig CDRs are linear sequences with specific binding properties. The high frequency of peptide binding in Ig CDRs may reflect their increased diversity by somatic mutation and clonal selection by antigens. Whether there may occur a proteolytic release of active fragments from immunoglobulins is a debatable hypothesis, that would be reminiscent of the extrinsic activity of Hb33-61 from bovine haemoglobin that displays antimicrobial activity against Gram-positive bacteria and fungi at µM concentrations, as well as the carboxy-terminal tripeptide (11-13, KPV) of α-MSH that inhibits *Staphylococcus aureus*, *C. albicans* and HIV-1 at picomolar concentrations [31]–[33].

A wide variety of organisms, from single-cell microorganisms, insects and other invertebrates, plants, amphibians, birds, fishes, and mammals, man included, produce antimicrobial, antiparasitic and antiviral peptides as part of their first line of defense [34]. As evaluated by searching the Blast (National Center for Biotechnology Information) data base, however, the sequences of the Ab CDRs investigated in this study appear to be characteristic of immunoglobulins and are very different from those of described natural peptides.

Here, it is clear that C7/pc42 H2 and HuA L1 are directly cytotoxic to tumor cells causing caspase-dependent apoptosis. Apparently, the pro-apoptotic activity of C7/pc42 H2 on melanoma cells depended on peptide binding to a surface receptor that specifically recognizes the C-terminal sequence. The C-terminal SYNQKFK peptide which is not apoptotic by itself competed with and inhibited the cytotoxicity of mAb C7/pc42 H2 in melanoma cells (data not shown). Amino acids K14 and F15 are crucial for the receptor-mediated mAb C7/pc42 H2 tumor cell cytotoxicity. C7 H3 but not C7/pc42 H2 competed with mAb C7 for binding to phosphatidylcholine, the probable ligand of polyreactive C7 on melanoma cells (unpublished results).

Apoptosis was characterized in both melanoma and HL-60 leukemia cells. H2 also inhibited endothelial cell growth on Matrigel™ suggesting that its *in vivo* antitumor effect could also involve inhibition of angiogenesis. Presently we show that both peptides in the more stable C-terminal amidated form were active against lung colonization by melanoma cells injected i.v. using a protocol of i.p. administration every-other-day for 11 days. A direct effect of peptides on tumor cells was needed for protection since the suspension of treatment resulted in a delayed death curve similar to the untreated control.

CDRs interference on HIV-1 replication was studied in PBMCs exogenously infected with different viral strains and in a more physiologic model of endogenously infected mononuclear cells. Presently, we show that some CDR synthetic peptides were able to inhibit R5 HIV-1 expression in naturally and exogenously infected PBMCs. We speculate that the interaction between CDR molecules and HIV-1 co-receptors CCR5 and CXCR4 in our culture systems were, probably, the limiting factor for HIV-1 replication. An alignment analysis (http://www.ebi.ac.uk/cgi-bin/clustalw) of the viral protein with the CDR sequences with high antiviral activity showed that important peptide motifs of HuA L3 (QRSNWPR) and of pc42 L1 (SKSVSTSG) were present in reverse transcriptase (RT) and Rev proteins, respectively. For HIV-1 inhibition, it has already been reported that many peptides could be competitive or non-competitive inhibitors of enzymatic activities by structure mimicry or by hindering the formation of enzyme-DNA/RNA complexes [35], [36].

Possible molecular targets in *Candida* to reactive pc42 L1 and L2 and HuA L3 CDRs could be β-1,3/β-1,6 glucans that neutralize these peptides as described for KP (data not shown). In the case of C7 H2, an N-terminal sequence in ALS3 is hypothesized to be a target based on its functional similarity with a peptide deduced by hydropathic complementarity to C7 H2 with codons read in the 3′–5′ direction [37].

Additional pathogenic agents and tumor cell lines obtained from type culture collections or isolated from clinical samples were also sensitive *in vitro* to the inhibitory activity of mAb C7, pc42 and HuA CDRs and engineered derivatives (unpublished data). They include eukaryotic and prokaryotic microorganisms such as *C. neoformans*, *A. fumigatus*, *S. prolificans*, *Pseudomonas aeruginosa*, methicillin-resistant *S. aureus*, viruses such as influenza A Ulster 73 (H7N1) and human NWS (neurotropic H1N1) and tumor cell lines HeLa (human cervix epitheloid carcinoma), CEM (human leukemia), Hs294T (human melanoma).

The easy production and the low cost of small sized synthetic peptides representing Ig CDRs may offer significant advantages compared to recombinant Abs or Ab fragments for the rational identification and design of novel antitumor, antimicrobial and antiviral biologically active and therapeutic compounds. As we demonstrated with KP and its D-isomeric form, such peptides can be easily engineered and stabilized against proteolysis by incorporation of non-natural amino acids or other modifications without affecting, or even enhancing, their activity [7], [38]. Peptide engineering and chemical optimization associated to new delivery mechanisms are expected to provide a new generation of therapeutic agents in parallel to the peptide vaccines that aim at protective immune responses [39].

## Materials and Methods

### Monoclonal antibodies and CDRs

Mouse mAb C7 (IgM), was produced as described elsewhere [11]. Single-chain variable region Ab fragments were obtained from the RNA extracted from the hybridoma cells secreting mAb C7 (scFv C7) by using the phage display methodology, as described [3]. After scFv C7 sequencing the CDRs of both light and heavy chains were identified [40]. All the CDRs of mAb C7 were chemically synthesized.

The mouse mAb pc42 (IgM), was selected from the Basic Local Alignment Search Tool (BLAST) of the National Center for Biotechnology Information data base, searching for short, nearly exact matches to the CDRs of mAb C7, as both mAbs shared the same CDRs H1 and H2. MAb pc42 is directed to the dominant epitope within a synthetic peptide model antigen (PS1CT3) including residues 28–42 of the large protein of the surface antigen of hepatitis B virus containing a B cell epitope (PS1) and the known T-helper lymphocyte epitope derived from the circumsporozoite protein of the malaria parasite *P. falciparum* (CT3) [15]. The hybridoma secreting mAb pc42 was kindly provided by Dr. Kanury V.S. Rao (International Center for Genetic Engineering and Biotechnology, New Delhi, India). As only the sequence of the heavy chain with the corresponding CDRs (H1, H2, H3) had been defined and published for mAb pc42, the genes encoding its light chain were cloned and sequenced and the CDRs (L1, L2 and L3) identified according to the procedures described for mAb C7.

Human mAb HuA (IgM) was secreted by a human-mouse trioma cell line which was a cloned Epstein Barr virus-transformed B cell line stabilized by fusion with the human-mouse fusion partner SBC-H20. The V_H_ and V_L_ chain cDNAs of HuA had been sequenced [16]. This Ab is representative of Abs widely spread in the population and all its CDRs differed from those of mAb C7 and mAb pc42. MAb HuA is specific for the most frequent difucosylated human blood group A substances. All the CDRs of mAb pc42 and mAb HuA were chemically synthesized. The mAb C7/pc42 H2 and mAb HuA L1 scrambled peptides had the sequences: QYKISCNKYTGSFNY and YARQVSSALSS, respectively.

### Engineering and evaluation of selected mAb C7 CDRs

The CDRs shared by mAb C7 and mAb pc42 (H1 and H2) showing antimicrobial, antiviral and/or antitumor activity *in vitro* or *ex vivo* were engineered by alanine-scanning. The alanine substituted derivatives (*asd*), defined according to the position held by the alanine-substituted aminoacid, were tested in the same biological assays to critically establish the functional relevance of each residue [5]. *In vitro* microbicidal activity of CDR peptides and selected *asd* against *C. albicans* UP 10 (clinical isolate from the fungi collection of the University of Parma) and *C. albicans* NCPF 3153 (National Collection of Pathogenic Fungi, Bristol, UK) were used for the *in vitro* experiments.

The microbicidal activity of synthetic CDRs against *C. albicans* strains was preliminarily evaluated by testing 100 µg/mL of each synthetic peptide by colony forming unit (CFU) assays as previously described [5]. Briefly, 10 µL of a distilled water suspension containing ∼5×10^4^/mL germinating yeast cells were added to 90 µL of distilled water containing the synthetic peptide to be tested. Yeast suspensions were incubated at 37°C for 6 h. After incubation, the cell suspensions were plated on Sabouraud dextrose agar, incubated at 30°C and observed for CFU determination after 48–72 hours. Each assay was carried out in triplicate. MAb CDR synthetic peptides exhibiting candidacidal activity at 100 µg/mL, and *asd* of mAb C7/pc42 H2, selected for the highest candidacidal activity, were further tested to determine EC_50_ values. EC_50_ was calculated by nonlinear regression analysis using Graph Pad Prism 4.01 software, San Diego, CA, USA.

### *In vivo* evaluation of anti-*Candida* activity of CDR peptides

Animal experiments were approved by the Institutional Review Board of the School of Medicine and Odontology at the University of the Basque Country, Spain. Female BALB/c mice, 8-weeks old (11–12 animals/group), were infected intravenously with 5×10^5^ *C. albicans* NCPF 3153 yeast cells suspended in 0.1 mL saline. Mice were treated i.p. with 200 µg of mAb C7 L1 and mAb C7/pc42 H1 and H2 for 3 days starting on day 0, and 4 h after the fungal challenge, and at 24 and 48 h thereafter. Control mice were injected with saline (same treatment schedule as those treated with the CDRs). Protection was evaluated by monitoring animal survival for 20 days. The mean survival time and numbers of CFU of *C. albicans* in infected tissues were calculated as reported previously [14].

### *Ex vivo* and *in vitro* evaluation of the inhibitory activity of CDR peptides and selected *asd* against HIV-1

Assessment of *ex vivo* antiviral activity of synthetic CDRs and *asd* of mAb C7 H1, selected for the highest antiviral activity in HIV-1 endogenous infection was performed as previously described [10], [41]. Briefly, for endogenous HIV-1 replication assays peripheral blood mononuclear cells (PBMCs) from R5 HIV-1-infected patients characterized by high viral RNA titers in plasma and cell-associated viremia were cultured in 96-well plates at 1×10^6^ cells/mL in RPMI 1640 medium with 10% fetal calf serum (FCS) and 20 U/mL of rIL-2. Exogenous rIL-2 was added every 3–4 days. Cultures were treated with synthetic mAb CDRs and mAb C7/pc42 H1 *asd* at 10 µg/mL during the time of culture. Virus production on days 5 and 10 was assayed in the supernatants using ultrasensitive Alliance® HIV-1 p24 ELISA kit (Perkin Elmer); p24 Ag in the supernatants of untreated cultures corresponded to 100% of viral production. For exogenous infection assays, PHA-stimulated PBMCs obtained from healthy individuals were infected with BaL (R5) or IIIB (X4) HIV-1 strain at a median tissue culture infective dose (TCID_50_) of 500 TCID_50_/ml. After 2 h of adsorption, the cells were washed, suspended at 2×10^5^ cells/ml in medium and cultured in 96-well cultures plates. At time zero of infection, synthetic peptides were added to the cultures at 10 µg/ml and were maintained throughout the experiment. All assays were performed in triplicate. Virus production was assayed 10 days after infection in the supernatants of HIV-1-infected PBMCs by p24 assay.

### Tumor cell lines and cell culture

Murine B16F10-Nex2 melanoma cells (cloned at the Experimental Oncology Unit, Federal University of São Paulo, UNIFESP), and human SKmel28 and SKmel25 human melanoma cell lines originally obtained from the Memorial Sloan Kettering Cancer Center, New York, were cultured at 37°C, under humid atmosphere and 5% CO_2_, in RPMI-1640 medium with 10 mM N-2-hydroxyethylpiperazine-N2 ethanesulphonic acid (HEPES), 24 mM sodium bicarbonate, 40 mg/L gentamycin, pH 7.2 and 10% FCS.

### *In vitro* tumor cytotoxic activity of CDR peptides and selected *asd*

Synthetic CDRs, scrambled peptides (controls), and *asd* of mAb C7/pc42 H2, selected for the highest cytotoxic activity, were diluted from 1 mM to 0.05 mM in RPMI with 10% FCS and incubated with B16F10-Nex2, SKmel28 and SKmel25 cells (5×10^3^ cells/well) in 100 µL per well for 12 h at 37°C. Each peptide was tested in triplicate. After 12 h, the cytotoxic activities of the peptides were determined by measuring cell viability by Trypan Blue exclusion. A 50% inhibition of cell growth was taken as a comparative index of cytotoxicity (EC_50_).

### DNA fragmentation assay

B16F10 cells were grown for 24 h in 12-well plates (10^5^cells/well), and then were further incubated for 12 h at 37°C with mAb C7/pc42 H2 (0.3 mM) and mAb HuA L1 (0.6 mM). Cells were then collected in phosphate-buffered saline-EDTA (PBS-0.02% EDTA), and centrifuged at 1,500 rpm. The pellets were suspended in 500 µL of TELT buffer [42] [50 mM Tris-HCl (Gibco), 4% Triton X-100 (LKB), 2.5 mM EDTA, pH 9.0 (Pharmacia) and 2.5 M LiCl (Merck)], pH 8.0. After centrifugation, equal volume of phenol (Gibco, pH 7.49–7.79) was added to the supernatant and centrifuged at 12,000 g/30 minutes. The aqueous phase was transferred to a new tube and equal volume of chloroform was added (Merck). After centrifugation (15 min, 12,000 g), equal volume of isopropanol was added to the pellet and cooled at −20°C for 24 h. The sample was centrifuged at 12,000 g/15 min, and the pellet washed with 500 µL of ethanol 70% (Merck). The dry pellet was resuspended in water with RNAse (20 µg/mL). Degradation of DNA in a ladder profile [43] was assessed in 0.8% Agarose gel electrophoresis (Pharmacia).

### *In vitro* Matrigel angiogenesis assay

Matrigel™ Matrix (B&D Biosciences, Bedford, MA, USA) was thawed on ice, added (10 µL/well) to coat 96-well plates, and allowed to polymerize for 1 h at 37°C. Human umbilical vein endothelial cells (HUVEC) (5×10^3^cells/well) were added alone or mixed with mAb C7 CDR peptides in 100 µL of RPMI medium supplemented with 0.2% of FCS in each well. All peptides were used at 1 mM, with the exception of mAb C7/pc42 H2 that was used at 50 µM. The plates were incubated at 37°C for 18 h and images were captured at 8× magnification with a Sony Cyber-shot camera coupled to a light microscope. The number of pro-angiogenic structures (closed rings formed at a given time by endothelial cell sprouting) was counted from 3 different wells, and the average value determined in each system [44], [45]. The assay was repeated at least three times.

### *In vivo* tumor protection assays with CDR peptides

All animal experiments were carried out using protocols approved by the Ethics Committee for animal experimentation of Federal University of São Paulo, Brazil. Thirty male C57BL/6 mice seven-to eight-week-old were injected i.v. with 5×10^5^ syngeneic B16F10-Nex2 viable cells in 0.1 mL for each mouse. For protection experiments three groups of 10 animals received on days 1, 3, 5, 7, 9, 11 after tumor cell challenge, i.p. doses of 250 µg of mAb C7/pc42 H2 and of mAb HuA L1, or PBS, at the same time periods, respectively. After 23 days, the lungs were collected from half of the animals of each group and inspected for metastatic colonization and the melanotic nodules were counted at 2× magnification. The survivals of remaining peptide-treated mice and controls were recorded till 40 days after i.v. challenge of tumor cells.

### Statistical analysis

The Kaplan-Meier log rank test was applied to survival data. Data on CFU in infected tissues were analyzed by Student's *t* test. *p* values of 0.05 were considered significant.
